# Supplementary material for: A Green, Rapid and Efficient Dual-Sensors for Highly Selective and Sensitive Detection of Cation (Hg2+) and Anion (S2−) Ions Based on CMS/AgNPs Composites
Source: Polymers (Basel). 2020 Jan 5;12(1):113. doi: 10.3390/polym12010113 (PMC7023171; doi:10.3390/polym12010113)
Supplement: Supplementary file 1 [file polymers-12-00113-s001.pdf]

Supplementary Material

A green, rapid and efficient dual-sensors for highly selective and sensitive detection of cation (Hg^{2+}) and anion (S^{2-}) ions based on CMS/AgNPs composites

Yun Xue[1], Lina Ma[1], Lei Zhang[1], Wanting Zhao[1], Zichao Li[2], Qun Li[1,*]

[1]*College of Chemistry and Chemical Engineering, Qingdao University, Qingdao 266071, China;*
xueyun@qdu.edu.cn (Y.X.); malina@qdu.edu.cn(L.M.); 2017020832@qdu.edu.cn (L.Z.); 2016020406@qdu.edu.cn (W.Z.)
[2]*College of Life Sciences, Qingdao University, Qingdao 266071, China;*
zichaoli@qdu.edu.cn (Z.L.);
** Correspondence: qunli@qdu.edu.cn (Q.L.); Tel.: +86-532-8595-0705 (Q.L.)*

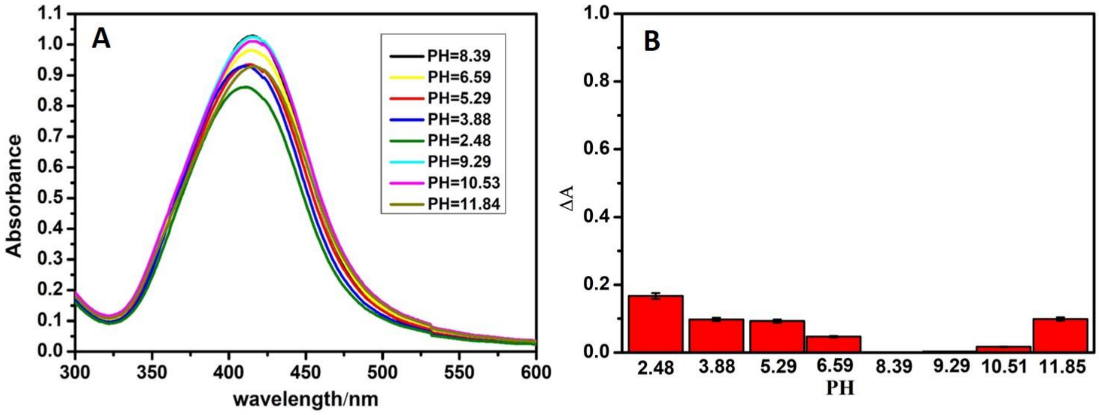

Fig. S1 (A) UV–vis spectra of CMS/AgNPs and (B) ΔA under different pH

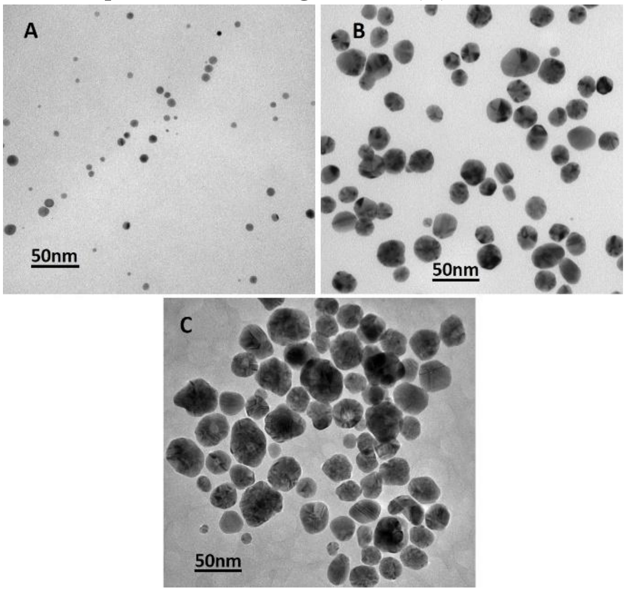

Fig. S2 TEM images of different reducing agent: (A) $NaBH_4$, (B) Sodium Citrate and (C) Glucose

Table S1 Comparison of the proposed Hg^{2+} detection method with different reducing agent

Reducing agent	Size of AgNPS/(nm)	Linear range/(μM)	LOD/(μM)
$NaBH_4$	～10	20-120	0.32
Glucose	～40	0-60	0.12
Sodium Citrate	～30	0-50	0.013
Vc	～20	0-50	0.018

Table S2 Comparison of the proposed Hg^{2+}/S^{2-} detection method with other reported methods

Methods	Probe	Ions	Linear range/(μM)	LOD/nm	Ref.
Colorimetric	β-CD AgNPs	Hg^{2+}	2.6-250	37.5	[1]
		S^{2-}	0.03-0.3	0.9	
Colorimetric	SSA-AgNPs	Hg^{2+}	0-5	14	[2]
		S^{2-}	0-0.8	4	
Colorimetric	AUNSs	Hg^{2+}	0.1-100	0.05	[3]
Electro chemistry	AgNPs	Hg^{2+}	5.0-755	0.19	[4]
Colorimetric	AgNPs	Hg^{2+}	0.5-5.0	58	[5]
Colorimetric	SA-AgNPs	Hg^{2+}	0.025-60	5.29	[6]
Fluorescence	NCDs-RhB@COF	Hg^{2+}	0.048-10	15.9	[7]
Electro chemistry	SPCE/Go/AuNPs	S^{2-}	3-40	300	[8]
Colorimetric	Cip-AgNPs	S^{2-}	-	16	[9]
Colorimetric	AgNPs	S^{2-}	3-40	300	[10]
Colorimetric	CMS/AgNPs	Hg^{2+}	0-50	18	This work
		S^{2-}	15-70	240	

1. Rajamanikandan, R. and M. Ilanchelian, *β-cyclodextrin functionalised silver nanoparticles as a duel colorimetric probe for ultrasensitive detection of Hg2+ and S2− ions in environmental water samples.* Materials Today Communications, 2018. **15**: p. 61-69.
2. Das, S., et al., *Sensitive and robust colorimetric assay of Hg2+ and S2− in aqueous solution directed by 5-sulfosalicylic acid-stabilized silver nanoparticles for wide range application in real samples.* Journal of Environmental Chemical Engineering, 2017. **5**(6): p. 5645-5654.
3. Chen, J.L., et al., *Determination of mercury (II) ions based on silver-nanoparticles-assisted growth of gold nanostructures: UV-Vis and surface enhanced Raman scattering approaches.* Spectrochim Acta A Mol Biomol Spectrosc, 2018. **199**: p. 301-307.
4. Eksin, E., et al., *Eco‐friendly Sensors Developed by Herbal Based Silver Nanoparticles for Electrochemical Detection of Mercury (II) Ion.* Electroanalysis, 2019. **31**(6): p. 1075-1082.
5. Ertürk, A.S., *Biosynthesis of Silver Nanoparticles Using Epilobium parviflorum Green Tea Extract: Analytical Applications to Colorimetric Detection of Hg2+ Ions and Reduction of Hazardous Organic Dyes.* Journal of Cluster Science, 2019. **30**(5): p. 1363-1373.
6. Faghiri, F. and F. Ghorbani, *Colorimetric and naked eye detection of trace Hg(2+) ions in the environmental water samples based on plasmonic response of sodium alginate impregnated by*

silver nanoparticles. J Hazard Mater, 2019. **374**: p. 329-340.

7. Guo, L., et al., *"On-off" ratiometric fluorescent detection of Hg(2+) based on N-doped carbon dots-rhodamine B@TAPT-DHTA-COF.* Spectrochim Acta A Mol Biomol Spectrosc, 2019: p. 117703.

8. Chen, Y.-H., et al., *Electrodeposited Ag, Au, and AuAg nanoparticles on graphene oxide-modified screen-printed carbon electrodes for the voltammetric determination of free sulfide in alkaline solutions.* Electrochimica Acta, 2016. **205**: p. 124-131.

9. Ayaz Ahmed, K.B., M. Mariappan, and A. Veerappan, *Nanosilver cotton swabs for highly sensitive and selective colorimetric detection of sulfide ions at nanomolar level.* Sensors and Actuators B: Chemical, 2017. **244**: p. 831-836.

10. Zhao, L., et al., *A Colorimetric Sensor for the Highly Selective Detection of Sulfide and 1,4-Dithiothreitol Based on the In Situ Formation of Silver Nanoparticles Using Dopamine.* Sensors, 2017. **17**(3): p. 626.
